# Unraveling the pathogenicity role of the novel compound heterozygous mutations of MED25 gene in a Chinese patient with BVSYS

**DOI:** 10.3389/fgene.2025.1654336

**Published:** 2025-08-20

**Authors:** Linbing Zou, Ruikang Qiu, Zhijun Dai, Yulei Li, Yunjiao Liao, Yan Zhou

**Affiliations:** ^1^ Hefei Maternal and Child Health Hospital Reproductive Medicine Center, Hefei, China; ^2^ Faculty of Medicine, Nursing and Health Sciences, Monash University, Clayton, VIC, Australia; ^3^ Xiangyang Central Hospital, Affiliated Hospital of Hubei University of Arts and Science, Xiangyang, China; ^4^ Department of Basic Medicine, School of Medicine, Jingchu University of Technology, Jingmen, China

**Keywords:** MED25, whole exome sequencing (WES), compound heterozygous mutation, c.1965+1dup, c.670C>G (p.R224G), gene splicing assay

## Abstract

**Introduction:**

Mediator of RNA polymerase II transcription subunit 25 (*MED25*), a crucial component of the transcriptional coactivator complex, plays a significant role in the transcription of most RNA polymerase II-dependent genes. Mutations in *MED25* have been linked to various genetic syndromes, including Basel-Vanagaite-Smirin-Yosef Syndrome (BVSYS) and Intellectual Disability (ID). This study elucidated the molecular mechanism through which compound heterozygous mutations in the *MED25* gene impaired pre-mRNA splicing, ultimately causing BVSYS.

**Methods:**

Whole exome sequencing (WES) was performed to identify genetic variants, followed by Sanger sequencing for validation. Clinical data were correlated with established *MED25*-related syndrome phenotypes. Bioinformatics tools were utilized to predict splicing effects and protein structural alterations. Functional characterization involved in vitro minigene splicing assays for the c.1965+1dup mutation and RT-PCR analysis of patient-derived transcripts, while the impact of p.R224G was assessed through protein structure modeling.

**Results:**

The proband presented with clinical manifestations such as cognitive impairment, language difficulties, intellectual disability, and microcephaly. The study identified a compound heterozygous mutation in the *MED25* gene (NM_030973.4), consisting of c.670C>G (p.R224G) and c.1965+1dup, which was associated with the observed clinical phenotype. Bioinformatics analysis and in vivo/in vitro splicing assays demonstrated that the c.1965+1dup mutation disrupts *MED25* pre-mRNA splicing, whereas the c.670C>G (p.R224G) variant does not exhibit this effect. However, bioinformatics analysis suggested that the mutation c.670C>G (p.R224G) may affect gene function by altering the structure of the *MED25* protein.

**Conculsion:**

These findings demonstrated that two mutation sites identified in the *MED25* gene in this case are pathogenic or likely pathogenic and may be associated with the clinical phenotype of the proband in this study.

## Graphical Abstract

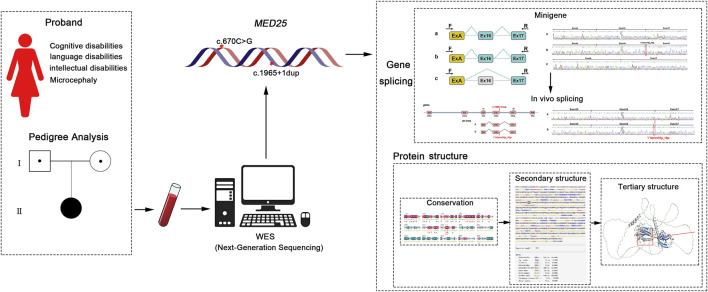

## 1 Introduction

The *mediator complex subunit 25* gene is located on *19q13.33* and encodes a component of the transcriptional coactivator complex termed the Mediator complex, mediator of RNA polymerase II transcription subunit 25 (MED25). This complex is required for transcription of most RNA polymerase II-dependent genes (Richter et al., 2022). MED25 plays a role in chromatin modification and in preinitiation complex assembly. Mutations in *MED25* are associated with Basel-Vanagaite-Smirin-Yosef Syndrome (BVSYS) and Intellectual disability (ID) (Figueiredo et al., 2015; Ba et al., 2015; Mai et al., 2021; Haynes et al., 2020; Nair et al., 2019). Among them, BVSYS and *MED25* variants are most closely related.

BVSYS was first discovered in 2015 (Figueiredo et al., 2015). In the same year, Basel-Vanagaite further described patients with more complex phenotypes (Figueiredo et al., 2015; Ba et al., 2015; Mai et al., 2021; Haynes et al., 2020; Nair et al., 2019). In addition to global developmental delay/intellectual disability, there are also multiple Congenital anomalies. So far, BVSYS was defined as a new identifiable syndrome characterized by global developmental delay/intellectual disability and craniofacial, neurological, ocular, and cardiac abnormalities. This study presents a case exhibiting clinical symptoms of cognitive impairment, language difficulties, intellectual disability and microcephaly. Whole exome sequencing (WES) revealed compound heterozygous mutations in the *MED25* gene, respectively c.670C > G (p.R224G) inherited from the father and c.1965 + 1dup inherited from the mother, which never been reported before. Given that the *MED25* gene is closely associated with various biological functions and may influence neurodevelopment, we conducted research to investigate the impact of two mutation sites on *MED25* gene splicing and protein structure. This study aims to clarify the pathogenicity of these variants of *MED25* and their relationship with the clinical phenotype of the proband.

In the present study, a comprehensive analysis of the clinical phenotypes of the study patients was conducted to elucidate the pathogenicity of the novel mutation sites identified in the proband. Initially, we employed bioinformatics to assess the potential effects of mutation sites on *MED25* gene splicing. Subsequently, the bioinformatics predictions were validated through both *in vitro* and *in vivo* gene-splicing assays. Additionally, we conducted further analysis of the mutation sites using protein structure prediction software to evaluate their impact on MED25 protein structure and stability.

## 2 Materials and methods

### 2.1 Samples, ethic approve and consent to participate

The samples used in this study were peripheral blood obtained from proband and their parents. The protocols for this study were evaluated and approved by the Ethics Committee of Xiangyang Central Hospital. Informed consent forms for WES analysis were signed by the parents of the proband, specifically for the purpose of this research.

### 2.2 Cells culture

The HEK293T and HeLa cell lines were purchased from the China Center for Type Culture Collection (CCTCC, China). The cells were cultured with the high-glucose Dulbecco’s Modified Eagle Medium (DMEM; Gibco, USA) containing 10% fetal bovine serum (Gibco, USA) and 1% penicillin-streptomycin (Gibco, USA), in a constant temperature incubator at 37 °C and 5% CO_2_ with saturated humidity.

### 2.3 Whole exome sequencing (WES)

WES was performed by inputting 150–300 ng of genomic DNA from each sample, which was extracted from peripheral blood samples of the proband and her parents according to the manufacturer’s instructions (MagPure Buffy Coat DNA Midi KF Kit, MAGEN). Exome capture using the MGIEasy Exome Capture V4 Probe (MGI) was followed by paired-end read sequencing (2 × 100 bp read length) on the MGISEQ-2000 platform with an average depth of ≥100-fold. WES and related data analysis were performed by Hangzhou Bosheng Medical Laboratory.

### 2.4 Bioinformatics analysis

Bioinformatics analysis was performed to investigate the effect of these variations on the *MED25* gene at two levels of regulation, the mRNA transcription level and the protein translation level. PolyPhen-2 (http://genetics.bwh.harvard.edu/pph2/index.shtml) was applied for the conservation analysis of genetic mutations. HSF (https://hsf.genomnis.com/login), RDDC^SC^ (https://rddc.tsinghua-gd.org/search-middle?to=SplitToolModel), and SpliceAI (https://spliceailookup.broadinstitute.org/) algorithms are used to predict whether mutations affect the splicing of *MED25* mRNA.

First, the InterPro (http://www.ebi.ac.uk/interpro/search/sequence/#opennewwindow), UniProt (https://www.uniprot.org/), and NCBI (https://www.ncbi.nlm.nih.gov/) databases were utilized to predict the MED25 protein domain. Subsequently, Consurf (https://consurf.tau.ac.il/consurf_index.php) was employed to analyze the conservation of the mutation site. The effects of mutations on the secondary structure of the MED25 protein were assessed using the SOPMA database. Additionally, Alphafold3 (https://golgi.sandbox.google.com/) and Missense3D (http://missense3d.bc.ic.ac.uk/∼missense3d/) were used to predict the impact of mutations on the tertiary structure of the MED25 protein. Finally, a comprehensive analysis of the effects of mutations on the stability of the MED25 protein was performed using multiple databases, including DUET (http://biosig.unimelb.edu.au/duet/stability), DynaMut2 (https://biosig.lab.uq.edu.au/dynamut2/submit_prediction_mm), I-Mutant2.0 (https://folding.biofold.org/i-mutant/i-mutant2.0.html), SAAFEC-SEQ (http://compbio.clemson.edu/SAAFEC-SEQ/#started), and MUpro (https://mupro.proteomics.ics.uci.edu/).

### 2.5 Minigene splice assays (MSA)

To study the effect of variations on *MED25* gene splicing, we used reverse transcription combined with nested PCR to analyze the subjects’ periphery *MED25* ectopic transcript. For the *in vitro* splicing study, the minigene pcMINI-N/C-wt/mut and pcDNA3.1-wt/mut were constructed. Based on the mutation site, we devised two vector construction strategies to assess any abnormalities in the splicing patterns. For the mutation c.670C > G p. R224G, which is situated at position −19 of Exon 6, we inserted Exon 5 - Intron 5 - Exon 6 - Intron 6 - Exon seven into the pcDNA3.1 or pcMINI-N vectors. In contrast, for the mutation c.1965 + 1dup, located at the +1 position of Intron 16, we incorporated a segment of Intron 15 - Exon 16 - Intron 16 - Exon 17 into the pcMINI-C vector.

We used genomic DNA as the template for nested PCR. The nested PCR products of the second round were used as templates to amplify wild-type and mutant DNA fragments. Vector and fragments were digested, recovered, ligated, and transformed into colonies for PCR identification and Sanger sequencing.

The successfully constructed wild-type/mutant recombinant minigene were transfected into Hela or HEK293T cells. 48 h later, total RNA was extracted. After reverse transcription synthesis of cDNA, we used primers on both sides of minigene for PCR amplification. Agarose gel was used to detect the size of products.

### 2.6 Splice assay *in vivo*

In the investigation of the c.1965 + 1dup mutation in the *MED25* gene, we utilized the father’s blood sample as a control to assess whether the mutation present in the mother’s sample influenced mRNA splicing. Total RNA was extracted from the whole blood samples of the proband’s parents and subsequently reverse transcribed. The cDNAs from both the father and mother served as templates for PCR amplification. The PCR products were analyzed using agarose gel electrophoresis and Sanger sequencing.

## 3 Results

### 3.1 Proband

The proband is a girl who was admitted to Xiangyang Central Hospital in Hubei Province at the age of 3 years and 10 months due to cognitive, language, and intellectual disabilities, as well as microcephaly. Neither of the proband’s parents had relevant clinical manifestations.

Now, at the age of 6.5 years, the proband exhibits persistent growth retardation according to WHO growth standards, with a height of 1.0 m (−2.3 SD) and a weight of 15 kg (−1.8 SD). In terms of motor function, the patient exhibits significant limitations and is unable to independently perform actions such as running, jumping, or climbing stairs. Comparative assessment results indicate that since the initial evaluation, there has been moderate development in both motor skills and cognitive function. The patient’s language comprehension remains intact; however, verbal expression has been completely lost. Notably, epileptic seizures began at the age of 3.5, presenting as high-frequency paroxysmal activity. During the most recent 2-month observation period, three episodes have been recorded.

**FIGURE 1:**
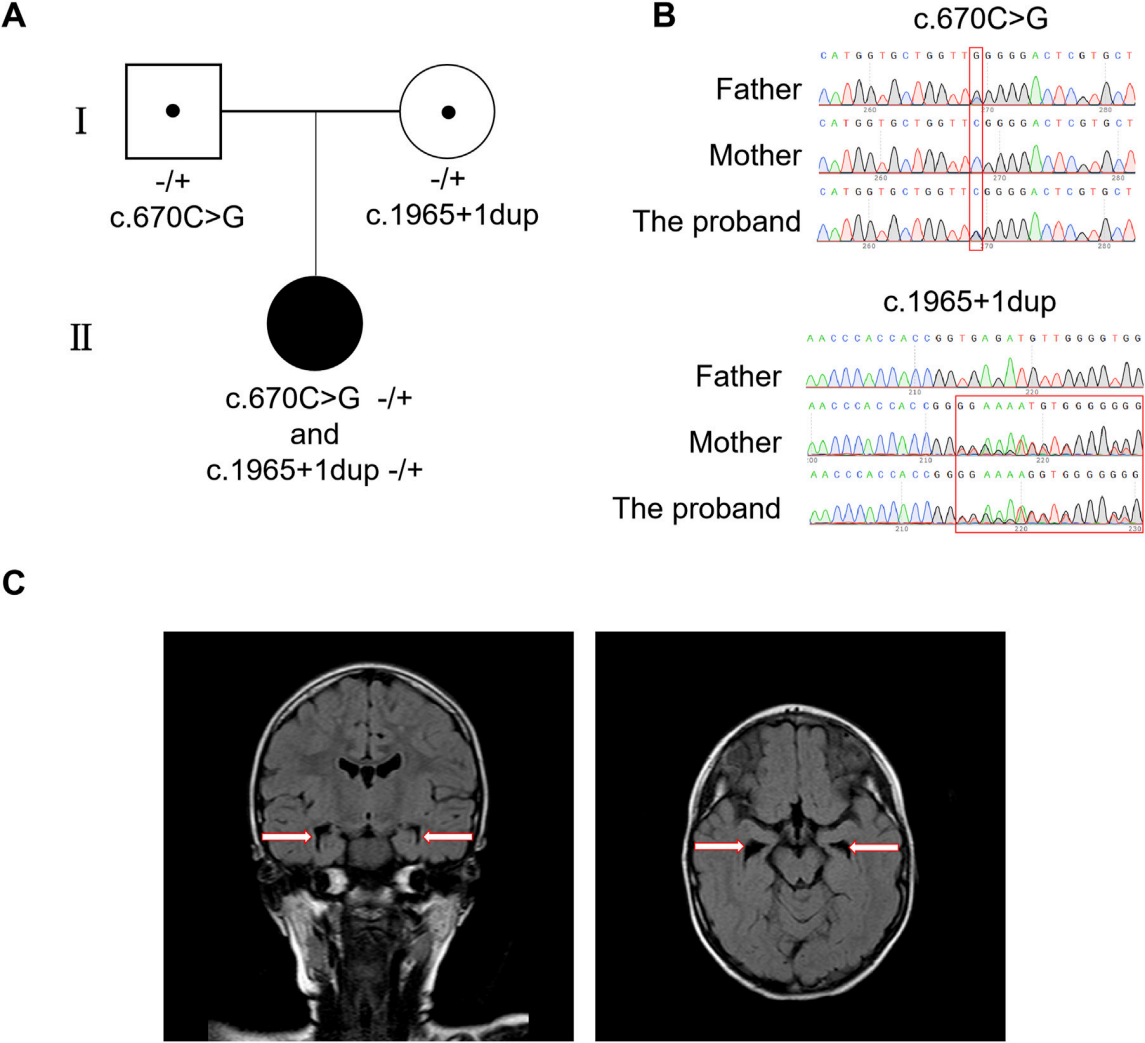
Identification of compound heterozygous mutations in *MED25* and the MRI results of proband. **(A)** The family tree. **(B)** Sanger sequences for the proband and her mother/father. **(C)** MRI results of the proband. The arrows point enlarge of the cavum septum pellucidum which indicates bilateral hippocampal volume loss.

The proband’s blood biochemistry and liver and kidney function tests revealed no abnormalities; however, the MRI and electroencephalogram indicated abnormalities. The brain MRI results showed scattered punctate lesions that appeared isointense on T1-weighted images and hyperintense on T2-weighted images in the subcortical white matter of both cerebral hemispheres, with high signal intensity on FLAIR sequences. Additionally, there was bilateral hippocampal volume loss with widening of the temporal horns, and the cavum septum pellucidum was present (Figure 1C). The EEG examination results indicated an abnormal electroencephalogram III (awake/sleep), with typical epileptic discharges detected in the central parietal and temporal brain regions and the anterior and posterior heads of the bilateral brain during the intermittent period, accompanied by tachycardia. A large number of low to mid-amplitude spine slow waves and multiple spine slow waves were observed in the bilateral central parietal and temporal regions, particularly in the left central parietal and temporal regions. A small amount of low to mid-amplitude spine slow waves and multiple spine slow waves were also seen in the bilateral posterior and anterior heads.

### 3.2 Mutation analysis by next-generation sequencing (NGS)

Using WES analysis, compound heterozygous variants of c.670C > G (p.R224G) and c.1965 + 1dup in the *MED25* gene (NM_030973.4) on chromosome 19 of the proband were detected, which have never been described before. These compound heterozygous variants were inherited from her father and mother respectively (Figure 1A). The analysis results indicated the proband’s mother is a heterozygous carrier of the *MED25* gene mutation c.1965 + 1dup, and the father is a carrier of the mutation c.670C > G (p.R224G) (Figure 1B). Moreover, the *MED25* gene is associated with Basel-Vanagaite-Smirin-Yosef syndrome, which is an autosomal recessive inheritance. Representative clinical features of BVSY include microcephaly and global developmental delay, which are consistent with the clinical phenotype of the proband. However, according to American Association for Human Genetics and Genomics (ACMG) guidelines, the *MED25* c.670C > G (p.R224G) variant was classified as a variant of uncertain significance (VUS), as it meets the PM2 criterion (the variant is absent in the gnomAD population database). The *MED25* c.1965 + 1dup variant was classified as a variant of uncertain significance (VUS) based on: PVS1 (a splice-site variant likely leading to aberrant splicing and potential functional impact on the gene) +PM2 (absent in the gnomAD population database).

### 3.3 c.1965+1dup mutation in *MED25* affected gene splicing both *in vitro* and *in vivo*

The WES analysis results indicated the c.1965 + 1dup variant of the *MED25* gene is a splice site mutation, which might lead to abnormal gene splicing and affect gene function. However, the impact of the variant c.670C > G (p.R224G) on gene splicing is unclear. Therefore, the *in vitro* minigene splice assays were firstly utilized to analyze whether the two variants detected in this study affect the alternative splicing of *MED25* gene, thereby further affecting gene function.

**FIGURE 2:**
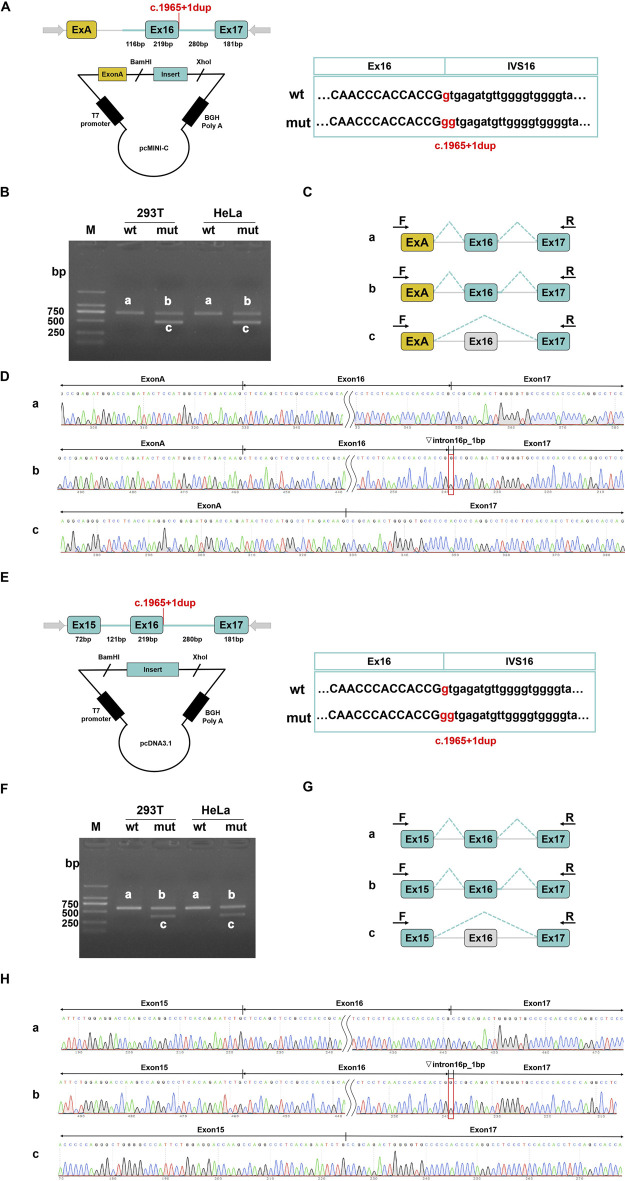
Results on *in vitro* splicing of variant c.1965+1dup that were tested in minigene splice assays. For the variant c.1965+1dup, the minigene pcMINI-C-wt/mut and pcDNA3.1-wt/mut were constructed. **(A–D)** pcMINI-C vector was utilized. **(E–H)** pcDNA3.1 vector was used. **(A,E)** The vector construction strategies are depicted respectively. The location of the variant is indicated in red. **(B,F)** HEK293T or Hela cells were transfected with WT or Mut constructs and then the total RNA was extracted to RT-PCR. The PCR products were applied to agarose gel electrophoresis as indicated. **(C,G)** A schematic overview of all the mRNA products that resulted from transfection with both constructs is visualized. **(D,H)** The results of Sanger sequencing of PCR products.

For each mutation site, we designed two sets of minigene vectors and validated them across different cell lines to ensure the objectivity and accuracy of our detection methods. *In vitro* MSA results indicated that the variant c.670C > G (p.R224G) did not disrupt the normal splicing of the MED25 gene (Supplementary Figure S3). In contrast, the variant c.1965 + 1dup affected normal splicing, resulting in two abnormal transcripts: retention of 1 bp in intron 16; skipping of exon 16 (Figure 2). The band intensity was scanned and analyzed using ImageJ to calculate the proportion of each transcript variant. The results showed that in the pcMINI-C vector, band b accounted for 53%, while band c made up 47%. In the pcDNA3.1 vector, band b represented 38% and 40%, whereas band c constituted 62% and 60%, respectively (Supplementary Figure S4). Both vectors demonstrated a higher proportion of the aberrantly spliced c band after mutation. Notably, the retention of 1 bp in intron 16 could lead to alterations in the subsequent reading frame, potentially resulting in a premature stop codon (PTC) in the 3′UTR region. Meanwhile, the skipping of exon 16 did not alter the reading frame but might result in the internal deletion of 73 amino acids in the protein.

**FIGURE 3:**
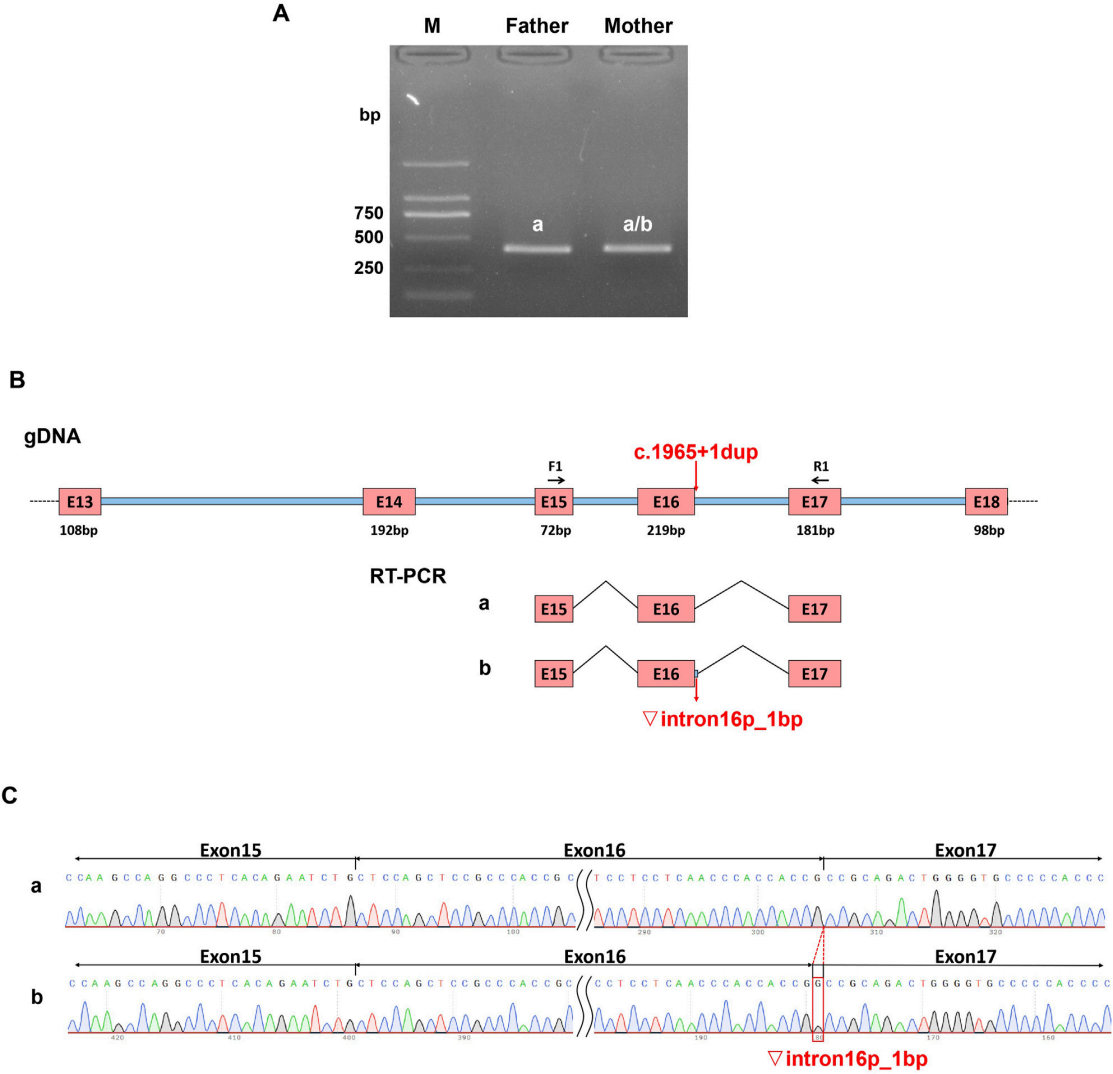
Effect of variant (C)1965+1dup on *in vivo* splicing of *MED25* gene. Total RNA was extracted from the whole blood samples of the proband’s parents and then reverse transcribed to get the cDNA as templates for PCR. **(A)** The PCR products were applied to agarose gel electrophoresis. **(B)** The schematic overview of the PCR products. **(C)** The Sanger sequencing results of PCR products.

To further confirm that the variant c.1965 + 1dup can cause abnormal splicing of MED25 mRNA *in vivo*, we conducted an *in vivo* splicing experiment. Using a sample from the proband’s father as a control, we assessed whether the mutation in the sample of proband’s mother affected mRNA splicing. PCR amplification was performed using the cDNAs from both the father and mother as templates. The results of the electrophoresis test indicated that both the father and mother exhibited a single band (Figure 3A). The gel electrophoresis bands were subsequently subjected to Sanger sequencing, revealing that the father’s band corresponded to the expected size of the normal splice variant, measuring 393 bp, while the mother’s sequencing results displayed a set of peaks. Consequently, the mother’s PCR product was purified and subjected to TA cloning followed by Sanger sequencing. The results indicated the presence of two splice variants: the normal splice variant and a variant with a retention of 1 bp on the left side of intron 16 (Figures 3B,C).

Here, we demonstrated through both *in vivo* and *in vitro* splicing experiments that the variant c.1965 + 1dup can disrupt the normal splicing of *MED25* mRNA, while, the variant c.670C > G (p.R224G) does not impact gene splicing.

### 3.4 c.670C>G p.R224G variant affected the structure of MED25 protein

**FIGURE 4:**
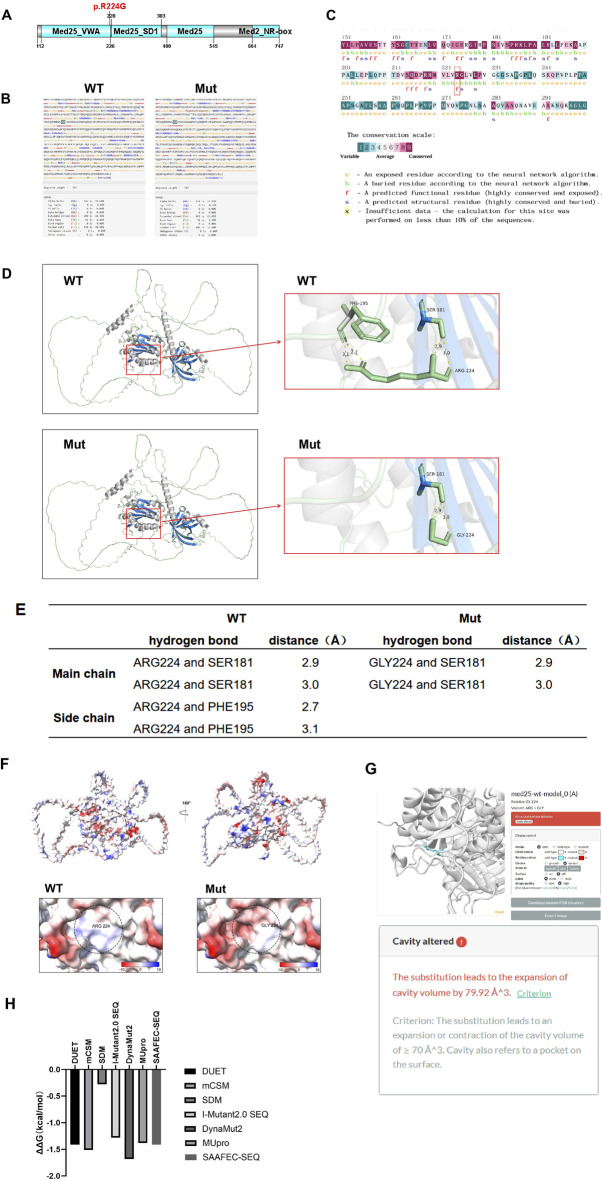
Effects of variant (C)670C>G p. R224G on MED25 protein. **(A)** NCBI prediction result of the location of the mutation site. **(B)** SOPMA analysis results of the impact of the mutation on the secondary structure of MED25. **(C)** The results of conservation analysis. The red box shows the mutation site. The conservation scale is measured from point 1 to 9. With the point increasing, the conservation level is increasing. **(D,E)** Alphafold3 modeling and PyMOL mapping analysis results of the impact of mutation on the hydrogen bond structure of MED25 protein. **(F)** ChimeraX analyzes the effect of mutation on the surface electrostatic potential of MED25 protein molecules. Blue represents the positive charge distribution area, red represents the negative charge distribution area, and white represents the uncharged area. **(G)** The prediction results of the damaging effect of mutation on the tertiary structure of MED25 using Missense3D. **(H)** Analysis results of the impact of mutation on the stability of MED25 protein. The output results are expressed as ΔΔG (kcal/mol). Negative values represent reduced protein stability, suggesting that the mutation affects protein stability.

Although the results of the *in vitro* minigene splice assays indicated that the variant c.670C > G (p.R224G) has no effect on the splicing of the *MED25* gene, the NCBI prediction results showed that the mutation at position 224 is located in the Med25_VWA functional domain (Figure 4A), and the amino acid sequence is highly conserved. Therefore, this missense mutation is likely to affect the structure and function of MED25 protein.

In order to clarify whether the variant c.670C > G (p.R224G) has an impact on the MED25 protein structure, we constructed the secondary and tertiary structures of the mutated MED25 protein through bioinformatics methods and analyzed them. SOPMA database analysis results showed that the proportions of secondary structures such as Alpha helix (Hh), Extended strand (Ee), and Beta turn (Tt) change after mutation. Among them, Hh accounted for 19.68% before mutation and increased to 21.95% after mutation. The proportions of Ee and Tt decreased after the mutation, from 14.59% to 6.83% before the mutation to 13.65% and 5.49% respectively (Figure 4B). This suggests that mutation could affect protein secondary structure.

Consurf was utilized for conservation analysis, and the analysis results showed that the wild-type amino acid at position 224 is highly conserved in terms of exposure and functionality of the protein (Figure 4C), indicating that this mutation is likely to affect the tertiary structure of the protein. So we analyzed the effect of mutation on the tertiary structure of MED25 protein subsequently. Through Alphafold 3 modeling and PyMOL mapping analysis, it was found that the mutation did not affect the structure of the MED25 main chain. However, when ARG224 is mutated into GLY224, the hydrogen bond formed between the side chain and the original PHE195 position is broken (Figures 4D,E), thus affecting the structure of the MED25 side chain. Using the Adaptive Poisson-Boltzmann Solver (APBS) in ChimeraX for mapping analysis, the results indicated that the mutation also has a certain impact on the surface electrostatic potential of the MED25 protein molecule (Figure 4F). Subsequent Missense3D prediction results suggested that the c.670C > G (p.R224G) mutation could lead to cavity alteration, which would have a destructive effect on the tertiary structure of MED25 (Figure 4G).

Finally, we analyzed the impact of the 224-position mutation site on the stability of MED25 protein through multiple software such as DUET, MUpro, DynaMut2, SAAFEC-SEQ and I-Mutant2.0 SEQ. The negative ΔΔG values across all algorithms suggest that the mutation reduces MED25 protein stability. Take mCSM or DUET for instance, a ΔΔG below −0.5 kcal/mol is indicative of high instability. As shown in Figure 4H, the mutation can significantly affect the stability of MED25 protein. It can be seen that the missense mutation c.670C > G (p.R224G) not only affects the structure of MED25, but also has a significant impact on protein stability.

## 4 Discussion

In eukaryotes, the MED complex participates in the regulated transcription of almost all RNA polymerase II-dependent genes (Harper and Taatjes, 2018). The MED complex is a multi-protein complex composed of more than 20 subunits (Nayak and Taatjes, 2022). Animal experimental studies have shown that some subunits in the complex can interact with specific transcription factors, thereby affecting development and/or differentiation through regulating the expression of different gene groups (Maalouf et al., 2024; Soutourina, 2018). Among them, the MED25 subunit plays an important role in the transcriptional activation of multiple transcription factors (Verger et al., 2013). These factors are involved in different development processes and control multiple metabolic pathways, including the development of motor and sensory neurons (Schiano et al., 2023; Koizumi et al., 2007) and cartilage formation (Nakamura et al., 2011; Akiyama and Lefebvre, 2011). Currently, several different site mutations on the *MED25* gene have been reported to be associated with genetic diseases. The variant c.418C > T (p.A140T) in *MED25* was reported to be related to intellectual disability (ID) (Figueiredo et al., 2015). The variant c.116A > G (p.T39C) of MED25 was reported in BVSYS (Ba et al., 2015). However, the proband in this report was detected by WES sequencing to have compound heterozygous variations in the *MED25* gene, namely, missense mutation c.670C > G (p.R224G) and splice site mutation c.1965 + 1dup, which have never been reported before. In this case, the clinical features of the proband were only cognitive-linguistic intellectual disability and microcephaly, and lacked other typical clinical features of BVSYS caused by *MED25* mutations. Considering that the clinical phenotype of this case may be caused by the mutations of *MED25*, a series of analyzes were conducted in order to clarify the pathogenicity of the novel mutations in the *MED25* gene of proband in the present study.

Among the various genetic mutations that can lead to hereditary diseases, pre-mRNA splicing mutations have garnered increasing attention from researchers (Lee and Rio, 2015). Given that pre-mRNA splicing is a critical step in eukaryotic gene expression, abnormal splicing can result in altered gene expression and modifications in protein function, which are common causes of disease (Love et al., 2023). Consequently, we first analyzed the potential effects of these two site mutations on splicing using HSF, RDDC^SC^, and SpliceAI. The results indicate that the likelihood of the missense mutation c.670C > G (p.R224G) impacting gene splicing is 50%, whereas the probability of the splicing site mutation c.1965 + 1dup affecting gene splicing is as high as 90% (Supplemental Data, Supplementary Figures S1, S2). Therefore, we employed a Minigene splicing assay to assess the effects of these two mutation sites on *MED25* gene splicing *in vitro*. The results confirm that the splicing site mutation c.1965 + 1dup does indeed affect the splicing of the *MED25* gene, potentially producing two distinct splicing variants: a 1 bp retention of intron 16 and skipping of exon 16; whereas the missense mutation does not influence gene splicing.

Considering that the minigene test is an *in vitro* assay, there are notable differences when compared to the actual biological *in vivo* environment. The *in vivo* gene-splicing test can directly reflect the manifestation of abnormal gene splicing within the organism. To further confirm the effect of the mutation c.1965 + 1dup on *MED25* splicing, we conducted *in vivo* gene-splicing assays. Using the proband’s father’s sample as a control, we assessed the impact of the mutation on *MED25* mRNA splicing in the mother’s blood sample. The results indicated that the mutation c.1965 + 1dup affects the splicing of the *MED25* gene *in vivo*, producing only one abnormal splicing product, a 1 bp retention at the left side of intron 16. This retention leads to early termination of MED25 protein translation, thereby impacting protein function. This observation suggests that while the minigene serves as an emerging and powerful tool for detecting pre-mRNA splicing abnormalities, it also has certain limitations. If conditions permit, it is advisable to conduct *in vivo* gene-splicing experiments to validate the findings of *in vitro* minigene analysis, in order to accurately present the effects of mutations on patients *in vivo*.

Gene mutations that affect splicing are among the common causes of abnormal protein expression and function; however, they are not the sole contributors, as mutations can also influence protein function or biological activity by altering its structure. In this study, we identified that the missense mutation c.670C > G (p.R224G) does not impact the splicing of the *MED25* gene. Additionally, NCBI predictions indicate that the mutation at position 224 resides within the von Willebrand factor type A (MED25 VWA) domain, which is crucial for the recruitment of MED25 into the Mediator complex (Ba et al., 2015). Consequently, this mutation is likely to affect the function of MED25 by modifying the protein structure. Therefore, we employed bioinformatics tools to predict the potential impact of these mutations on the structure of the MED25 protein.

The missense mutation c.670C > G (p.R224G) results in the substitution of ARG at position 224 of the MED25 amino acid sequence with GLY, leading to alterations in the proportions of structural elements such as alpha helices (Hh), extended strands (Ee), and beta turns (Tt), thereby affecting the protein’s secondary structure. Furthermore, Consurf analysis indicates that the amino acid at position 224 of the wild-type MED25 protein is highly conserved, suggesting that mutations at this site may impact the protein’s exposure and functionality. Such changes in protein exposure could directly influence its solubility and stability. Additionally, functional alterations in the protein may affect enzymatic activity, ligand binding capabilities, and protein-protein interactions. Therefore, we hypothesize that although the missense mutation c.670C > G (p.R224G) does not disrupt the splicing of the *MED25* gene, it is likely to induce changes in the protein’s biological activity. This hypothesis is supported by bioinformatics analyses, which reveal that the mutation from ARG to GLY at position 224 significantly alters the protein’s electrostatic potential and stability. Moreover, the mutation adversely affects the protein’s structural integrity. Consequently, we conclude that the missense mutation c.670C > G (p.R224G) impacts the function of the MED25 protein by altering its structure.

In this study, the frequencies of the two mutation sites in the MED25 gene are not included in the gnomAD database. However, the c.1965 + 1dup mutation in the *MED25* gene is classified as a splice site mutation, which may lead to abnormal gene splicing and affect gene function. We conducted *in vivo* and *in vitro* splicing experiments, alongside various bioinformatics prediction tools, to identify the pathogenicity of two variants of the *MED25* gene. Consequently, according to the ACMG guidelines, the splicing site variant c.1965 + 1dup had increased the PP3 evidence (The harmful probability predicted by the splicing prediction tool (SpliceAI/HSF) was greater than 90%), and had been upgraded to a pathogenic variant (PVS1 + PM2 + PP3); while the missense mutation c.670C > G had increased the PP3(Protein structure prediction is harmful and leads to a decrease in stability) + PM1(Located in the key functional domain (MED25_VWA domain), this domain mediates the recruitment of the Mediator complex (verified by UniProt/NCBD)) + PP4(The phenotype was core-matched with the MED25-related diseases (intellectual disability + microcephaly)) evidence, and was classified as a likely pathogenic variant (PM2 + PP3 + PM1 + PP4).

In conclusion, we identified compound heterozygous mutations, including a novel splice site mutation and a missense mutation, in the proband by applying NGS. Through bioinformatics and *in vitro*/*in vivo* splicing assays, the splice site mutation was proved to cause *MED25* gene function change *via* aberrant splicing and the missense mutation induce protein structure change leading to the dysfunction of MED25.

## Data Availability

The original contributions presented in the study are included in the article/Supplementary Material, further inquiries can be directed to the corresponding authors.
