# Supplementary material for: Unraveling the pathogenicity role of the novel compound heterozygous mutations of MED25 gene in a Chinese patient with BVSYS
Source: Front Genet. 2025 Aug 20;16:1654336. doi: 10.3389/fgene.2025.1654336 (PMC12405326; doi:10.3389/fgene.2025.1654336)
Supplement: Supplementary file 1 [file DataSheet1.docx]

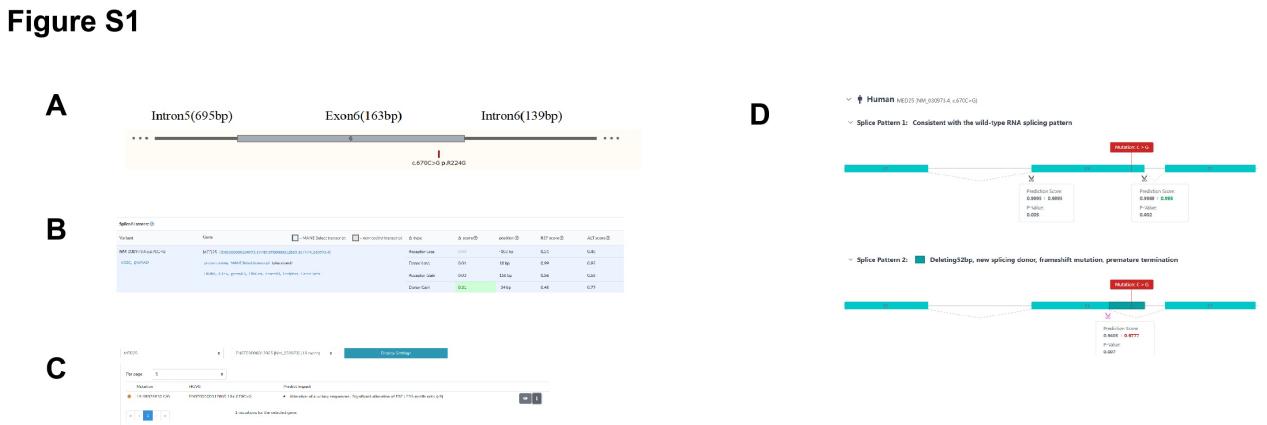


**Fig. S1 Prediction of the impact of mutation c.670C>G p.R224G on *MED25* gene splicing. A.** The location of mutation c.670C>G on *MED25* gene. **B.** The prediction result of SpliceAI. **C.** The prediction result of HSF. **D.** The prediction result of RDDC^SC^.


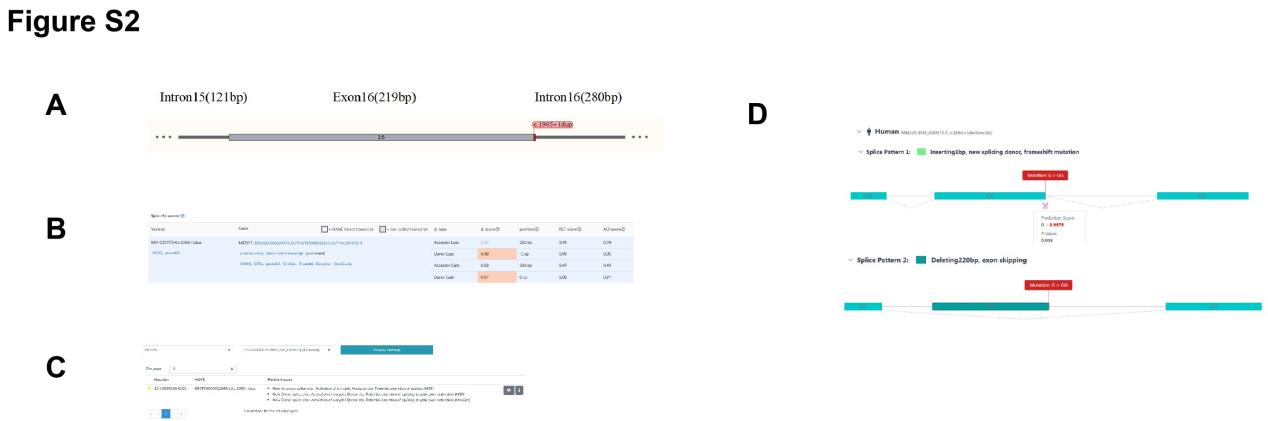


**Fig. S2 Prediction of the impact of mutation c.1965+1dup on *MED25* gene splicing. A.** The location of mutation c.1965+1dup on *MED25* gene. **B.** The prediction result of SpliceAI. **C.** The prediction result of HSF. **D.** The prediction result of RDDC^SC^.

Figure S3


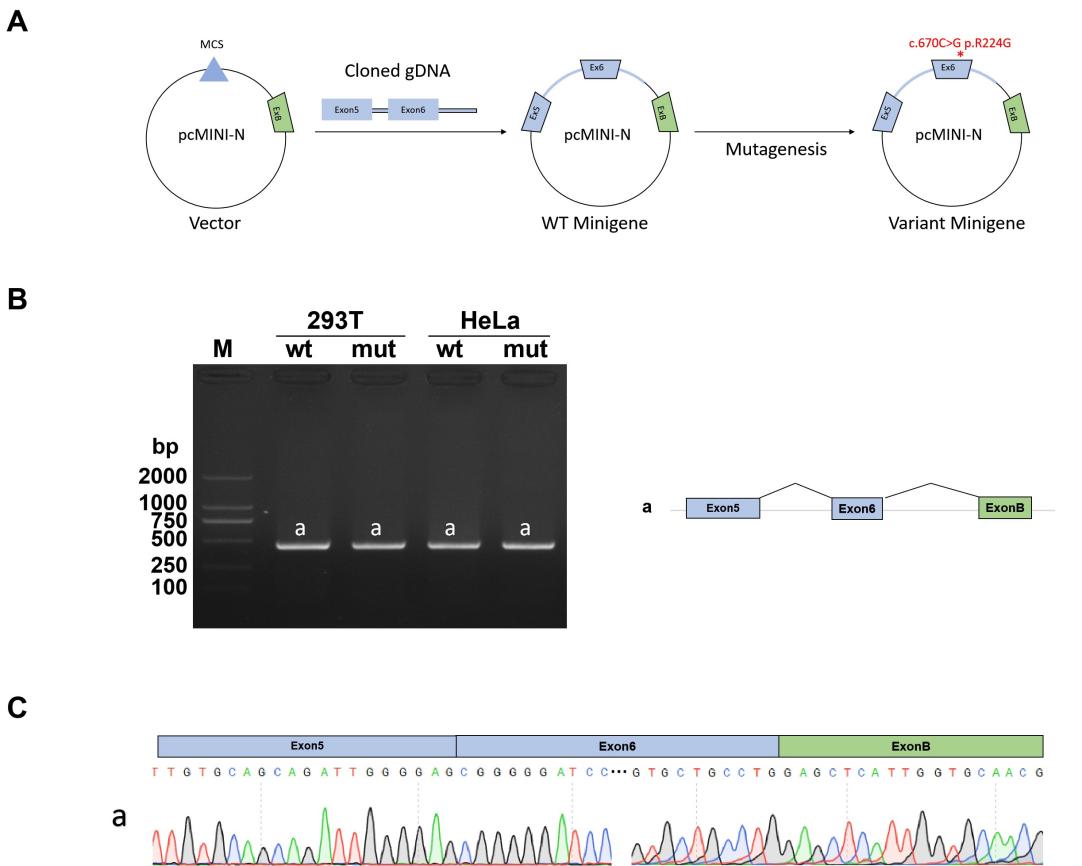


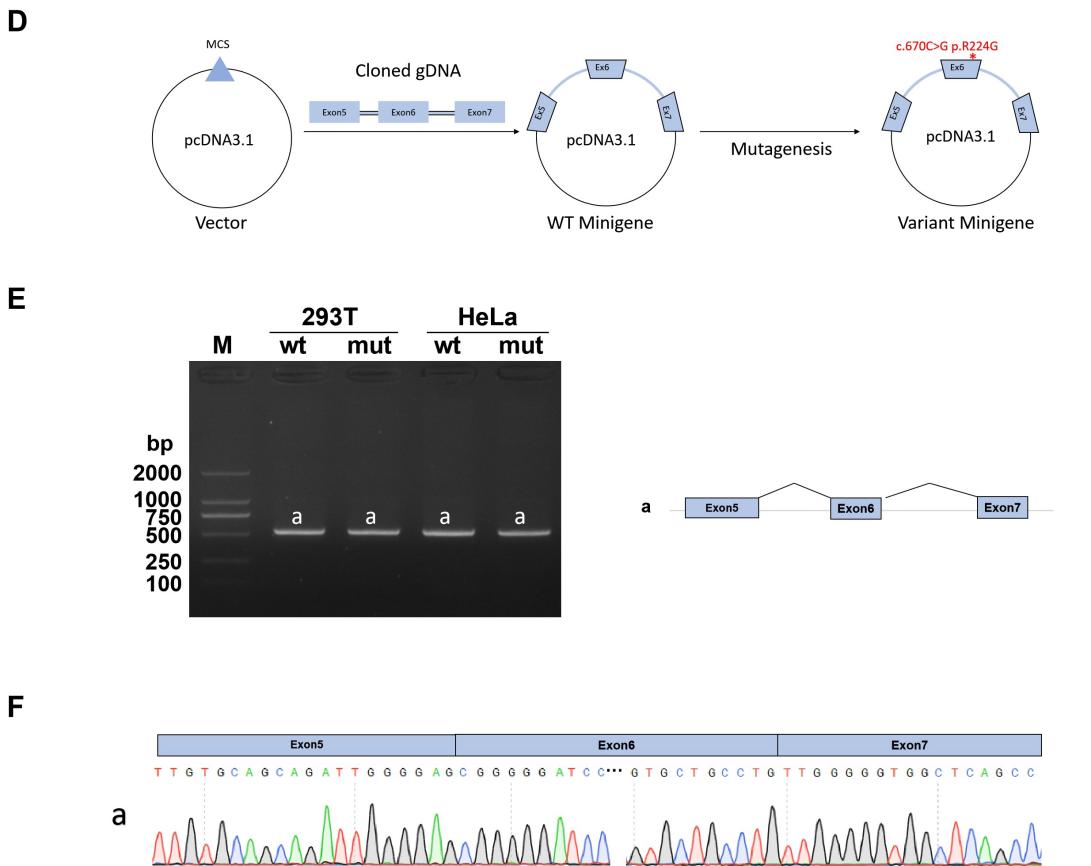


**Fig. S3** **Results on *in vitro* splicing of variant c.670C>G p.R224G that were tested in minigene splice assays.** For the *in vitro* splicing study of variant c.670C>G p.R224G, the minigene pcMINI-N-wt/mut and pcDNA3.1-wt/mut were constructed. **A-C.** pcMINI-N vector was used. **D-F.** pcDNA3.1 vector was utilized. **A and D.** The construction strategies of different vectors are depicted respectively. The location of the variant is indicated in red. **B and E.** HEK293T or Hela cells were transfected with WT or Mut constructs and then the total RNA was extracted and reverse transcripted into cDNA. The PCR products were applied to agarose gel electrophoresis as indicated. **C and F.** The results of Sanger sequencing of PCR products.

Figure S4


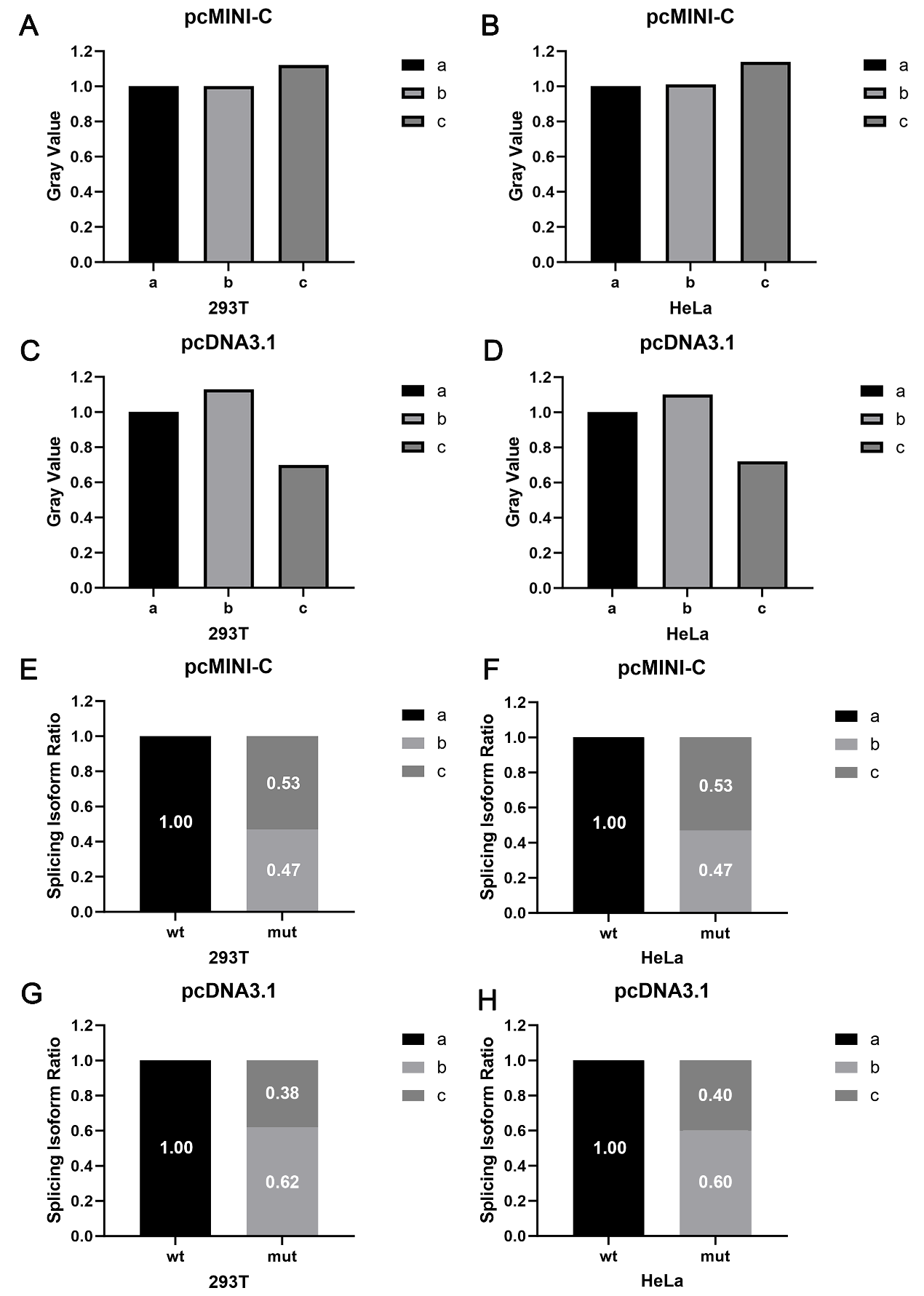


**Fig. S4 Quantitative analysis of c.1965+1dup splicing assay results. A-D**. Analysis results of the gray values of different bands; **E-H.** Analysis results of splice band ratios. Splicing isoform Ratio was calculated as[target band/total band].
